# Behaviour of a Preloaded Asymmetric Multi-Bolted Connection Under Cyclic Loads by Experimental Research

**DOI:** 10.3390/ma19071414

**Published:** 2026-04-01

**Authors:** Rafał Grzejda, Arkadiusz Parus, Konrad Kwiatkowski

**Affiliations:** Faculty of Mechanical Engineering and Mechatronics, West Pomeranian University of Technology in Szczecin, 70-310 Szczecin, Poland; arkadiusz.parus@zut.edu.pl (A.P.); konrad.kwiatkowski@zut.edu.pl (K.K.)

**Keywords:** multi-bolted connection, preload, cyclic loading, bolt force monitoring, resistance strain gauges

## Abstract

**Highlights:**

**What are the main findings?**

- Forces in the bolts remain stable within a range of ±2% of the initial preload of 22 kN for all cyclic loads.
- Bolts near the base of the connection show symmetric unloading due to prying action, while the other bolts exhibit slight overloading.
- No preload relaxation, loosening or fatigue-relevant stress amplitudes occur across nine test variants.

**What are the implications of the main findings?**

- Properly preloaded asymmetric multi-bolted connections are safe under operating cyclic loading without additional anti-loosening measures.
- Results provide an important quantitative benchmark for validating the systemic approach to modelling multi-bolted connections.
- The literature gap on multi-bolted connections has been filled with asymmetric geometry and loading.

**Abstract:**

This paper reports on experimental investigations of a preloaded asymmetrical multi-bolted connection under operating conditions. At the beginning of the tests, the bolts were preloaded in a predetermined optimal order, in three passes, monitoring the bolt force values by means of a calibrated measuring system with strain gauges. The main tests were carried out on an Instron 8850 testing machine. The connection was subjected to sinusoidal loads with amplitudes of 10 kN and 20 kN at various frequencies. The data obtained was collected and recorded using prepared measurement pathway components, and then processed in MATLAB/Simulink R2018b. The observed measurement results are depicted in graphs displaying the distribution of forces in bolts as a function of cyclically varying operating loads. We have demonstrated that the forces in bolts also vary cyclically relative to their initial preload values. Due to the asymmetry of the connection, this variation was different for individual bolts. The results show bolt force deviations within ±2% and confirm the stability of the connection under cyclic operating loads. The presented results complement the database of experimental results, on the basis of which we will validate the systemic approach to modelling multi-bolted connections.

## 1. Introduction

Multi-bolted steel and aluminium connections with preloaded bolts are among the most used nodes in mechanical and civil engineering, with which flange or lap joint systems are created [1,2,3]. Their proper functioning depends mainly on the behaviour of the bolt forces [4]. With cyclic loads applied to both single-bolted connections (SBCs) and multi-bolted connections (MBCs), the variability of these forces can be noticed [5,6,7]. Bolt forces tend to decrease or relax during cyclic loading, as evidenced in [8]. A lack of adequate mechanical protection can even lead to self-loosening of connections [9,10]. An overview of the causes and mechanisms of non-rotational and rotational loosening of the bolts was reported by Gong et al. [11]. Measures to prevent the loosening of MBCs subjected to dynamic loading include, but are not limited to, coating the bolts [12]. Furthermore, the cyclic tensile behaviour of preloadable bolts manufactured in the 10.9 grade of mechanical properties, commonly used for structural applications, was discussed by D’Aniello et al. [13]. This study provided valuable data on the monotonic and cyclic characteristics of high-strength bolts, but it was limited to individual fasteners without interaction in multi-bolted assemblies.

Testing of cyclically loaded MBCs usually consists of observing the deformation of the components of these connections during their operation. This leads to the determination of hysteretic stiffness curves and the formulation of conclusions regarding the considered connection. Selected experimental studies on this subject include the following papers. Zhou et al. [14] tested the bases of columns with T-stub connections. The structures proposed by them were assessed as useful for the seismic design of ductile steel moment frames. Their results added valuable insights into the design of seismic structures for ductile frames, but the tests were performed only on symmetric T-stubs and did not monitor bolt forces. Qualitatively similar results were presented by Latour et al. [15]. Piluso and Rizzano [16] developed rules for the degradation of stiffness and strength of bolted T-stubs subjected to cyclic loads as a function of the displacement amplitude required in any cycle and the energy dissipated in the previous load history. This work contributed significantly to the development of degradation modelling, but remained limited to symmetric connection configurations. Elghazouli et al. [17] investigated the cyclic behaviour of blind-bolted angle connections. Their tests demonstrated the importance of ensuring an adequate mechanism for transferring the compression forces to the column to achieve satisfactory performance of blind-bolted angle connections, but they omitted bolt force measurements and asymmetric loading. Nuñez et al. [18] analysed bolted beam-to-column connections with closed cross-sections with the aim of determining their ultimate behaviour. However, changes in the preload of bolts under cyclic operating loads were omitted in this work. Vatansever and Yardimci [19] reported the results of tests on the behaviour of a semi-rigid frame subjected to cyclic loads. Lemos et al. [20] described research on an innovative double-split tee connection under cyclic loading. These studies demonstrated practical seismic applications, but their scope was restricted to symmetric or innovative symmetric joints. McCrum et al. [21] determined the experimental cyclic performance of cold-formed steel bolted moment resisting frames. Orkun et al. [22] conducted tests of bolted stiffened end-plate and bolted flange-plate connections. They examined the effect of using different member profiles in connections on their performance. Hou et al. [23] carried out cyclic tests on bolted foundation connections for wide-flange columns. They identified the boundary conditions under which axially compressed bolted foundation connections maintain satisfactory energy dissipation performance. Tizani et al. [24] investigated the hysteretic performance and reliability of blind bolted connections to concrete-filled columns subjected to cyclic loading. Collectively, these works enriched the database on hysteretic behaviour, but they were all limited to conventional symmetric MBCs and did not include direct bolt force monitoring.

Testing of cyclically loaded MBCs less frequently relies on the observation of changes in bolt forces during connection operation. Kasai and Xu [25] presented a systematic test programme aimed at investigating the influence of multiple parameters on the inelastic cyclic behaviour of bolted angle connections. This programme added important parameter sensitivity data and observed preload loss, but was limited to symmetric angle connections. Lu et al. [26,27] determined the decrease in bolt preload under cyclic loading of reinforced legs in a retrofitted transmission tower. Their findings quantified preload reduction in retrofitted structures, but they addressed only specific tower geometries without general asymmetric cases. Chakherlou and Abazadeh [28] investigated the variability of bolt preload in a double-shear lap connection under cyclic loads. This study provided valuable interference-fit data; however, it examined only symmetric lap joints. Jaszak [29] predicted the durability of a gasket operating in a bolted flange connection subjected to cyclic bending by observing changes in bolt forces. The approach usefully linked bolt forces to gasket life, but was restricted to flange-type symmetry. In all the above-mentioned cases, bolt forces were measured using the strain gauge method (for review see [30]). There are also studies focusing on the observation of bolt failure mechanisms in bolted connections subjected to cyclic loads [31,32,33,34].

The results of the above review indicate that currently SBCs and conventional (i.e., usually symmetric) MBCs, such as flange connections (including T-stub and beam-to-column connections) or lap connections, are most frequently analysed. Their added value lies in hysteretic curves and seismic recommendations, but their common weakness is the lack of studies on connections with arbitrary bolt arrangements or those subjected to asymmetrically applied cyclic loads. To fill this gap, this paper describes the results of research on a multi-bolted connection characterised by geometric and load asymmetry. This gives the research a universal nature and makes it innovative compared to the studies described in the literature.

Since two states of load and deformation need to be taken into account in the case of MBCs [35], the asymmetric connection selected for testing was first preloaded and then subjected to various types of operating loads. The results of experimental studies on the preloading process of the multi-bolted connection have been reported in [36,37]. Tests of the same connection subjected to monotonic loads were the subject of paper [38]. An extension of these tests is the assessment of the cyclically loaded connection, which is presented in this paper. The results presented therein complement the database of experimental results, on the basis of which we will validate the systemic approach to MBCs modelling, the general principles of which are presented in [39,40]. The proposed systemic approach to modelling involves breaking down the connection components into separate subsystems and modelling them using any method. These subsystems include the pair of plates being joined, the interface layer between them, and the bolt assembly. We plan to write an article describing this approach in its entirety. It will cover the preload condition and the operating condition (caused by monotonic or cyclic forces).

## 2. Tested Multi-Bolted Connection and Bolt Calibration

Prior to the design of the test rig, the following criteria were specified:

- Rig will be used exclusively for measuring bolt force values;
- Outer dimensions of the complete connection will be derived from an analysis of the available strain gauges and the required cylindrical surface dimensions on the bolt shanks utilised in the connection;
- Components will be assembled using an odd number of fasteners;
- Connection will feature an asymmetrical contact surface between the assembled components.

**Figure 1:**
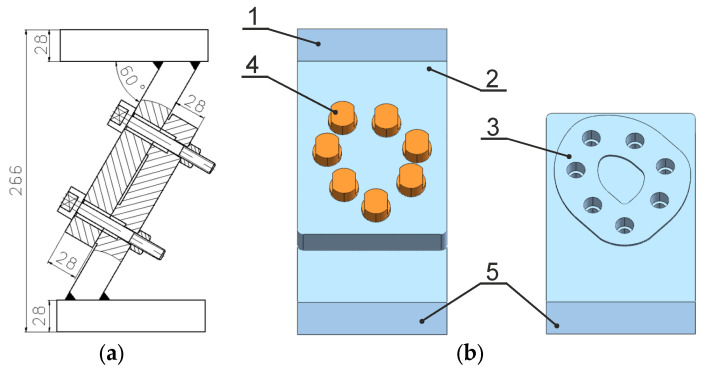
Analysed connection: (**a**) scheme; (**b**) model view (1—top plate; 2, 3—connection plates; 4—M10 × 1.25 bolt with a high hex nut; 5—base)—for comparison, see [38].

Taking the aforementioned criteria into account, a multi-bolted connection was designed and manufactured, the scheme of which is illustrated in Figure 1a. A simplified 3D model of the examined connection is presented using the MIDAS NFX 2023 R1 programme (MIDAS Information Technology Co., Ltd., Seongnam-si, Republic of Korea) in Figure 1b.

The analysed connection includes two plates (2) and (3) having a thickness of 28 mm, fixed by 7 M10 × 1.25 bolts (4) torqued with high hex nuts. The connection is deflected from the horizontal by 60 degrees. Plate (2) is welded to the top plate (1) and plate (3) is welded to the base (5). The examined connection is 266 mm high. The asymmetry of the connection results from the fact that the bolts are unevenly distributed on the asymmetric contact surface of the joined plates (as shown in Figure 1b) and from the odd number of bolts. The multi-bolted connection under investigation is deliberately asymmetric. As mentioned in the Introduction, the results of the experimental research are to be used to verify the correctness of the new method of modelling multi-bolted connections treated as a system. The purpose of this verification is to examine whether the systemic approach is universal enough to be applied to modelling a geometrically asymmetric connection subjected to a force acting in any direction (due to the deviation of the connection from the vertical, the bolts are subjected to loads in both the normal and tangential directions). The multi-bolted connection design discussed in this paper is intended to enable its testing under operating loads applied to the top plate (1) using an Instron 8850 servo-hydraulic testing machine (Instron GmbH, Darmstadt, Germany) [41,42]. Due to the deflection of the contact surface of the jointed plates from the horizontal plane, the connection may be subjected to both compressive and shear forces [43,44].

All plates in the connection were manufactured from 1.0577 non-alloy steel [45,46]. The bolts were manufactured in the 8.8 grade of mechanical properties, and the nuts in the 8 grade of mechanical properties [47,48,49]. The bolts were distributed in the connection in accordance with the guidelines provided in [50,51], but maintaining their non-regular spacing relative to each other.

Heat treatment was applied to the bolts to minimise hysteresis during calibration. Moreover, to minimise the impact of the number of contacts in the tested connection on modelling accuracy, no washers were used in the connection (as noted in the Introduction, the test results are planned to be used to verify the modelling of multi-bolted systems).

**Figure 2:**
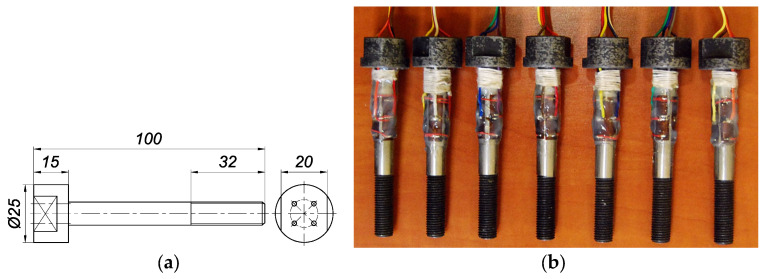
Bolt set used in the tests: (**a**) sketch of a single bolt; (**b**) general view—for comparison, see [38].

A sketch of a single bolt used in the tests is shown in Figure 2a, while a general view of the bolt set is illustrated in Figure 2b. The force changes in each bolt were measured using four Tenmex TFxy-4/120 strain gauges (Tenmex Electrical Resistance Tensometry Laboratory, Łódź, Poland) with two measuring ladders arranged perpendicularly [52]. They were attached to the outer part of the shank of each bolt in a full Wheatstone bridge system [53]. In order to bring out the data cables from the strain gauges, it was necessary to drill four 2 mm diameter holes in each bolt head. The location of the holes is shown in Figure 2a. Drilling these holes did not affect the mechanical properties of the bolts. The smallest cross-section along the length of the bolts remained unchanged and was equal to the cross-section of the thread core in the bolts. All bolts were custom-manufactured entirely by machining at the Centre for Advanced Manufacturing at the West Pomeranian University of Technology in Szczecin, Poland [54].

**Figure 3:**
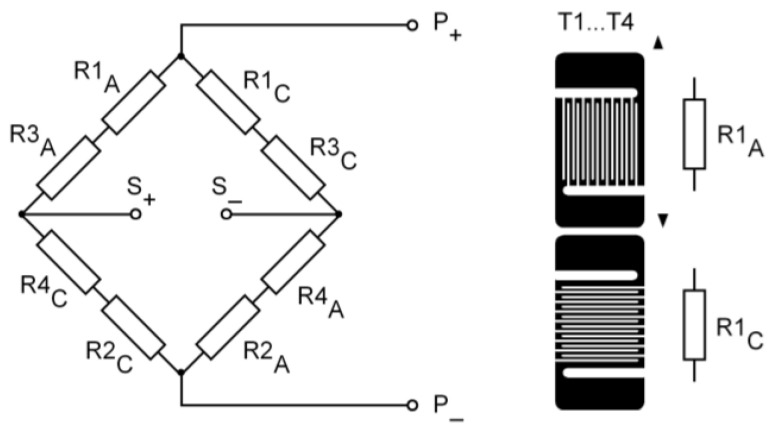
Diagram of a strain gauge system attached to a single bolt.

A diagram of the strain gauge system attached to a single bolt is additionally shown in Figure 3.

**Figure 4:**
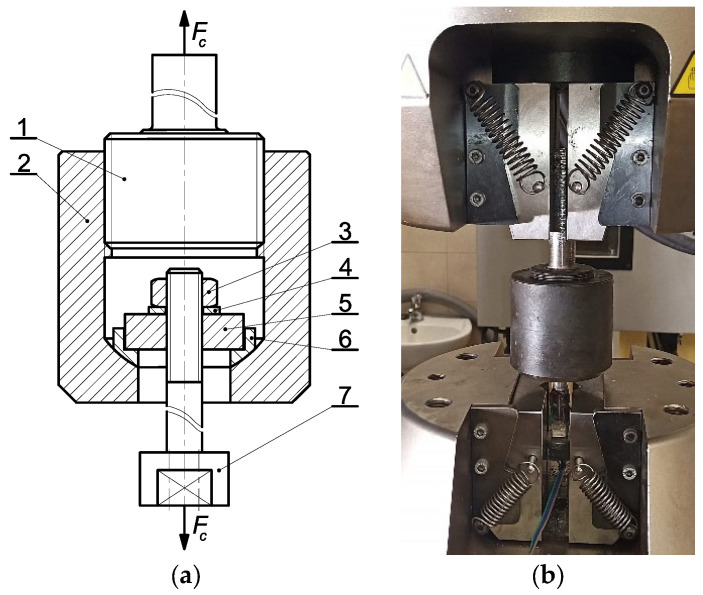
Bolt calibration equipment: (**a**) scheme (1—special bolt; 2—holder; 3—nut; 4—washer; 5—plate; 6—spherical sleeve; 7—tested bolt), (**b**) general view (*F_c_* denotes the calibration axial force) [38].

Each bolt was calibrated using an Instron 8850 servo-hydraulic testing machine (Instron GmbH, Darmstadt, Germany) equipped with a specially designed fixture (Figure 4).

Bolts (7) were sequentially positioned in the ball socket of the holder (2) using a set of the plate (5) and ball sleeve (6). The bolt was loaded with the hex nut (3) in the 10 grade of mechanical properties. The washer (4) was used between the nut (3) and the plate (5). This created a ball joint, ensuring the axial load of the bolt (7). Calibration was carried out after installing the holder (2) using the special bolt (1) in the top jaws of the testing machine. Meanwhile, the head of the tested bolt (7) was mounted on its cylindrical surface in the bottom jaws of the testing machine.

As a result of calibration, linear characteristics [55] were obtained for each bolt without observing hysteresis. They were used to identify the forces acting on the bolts by means of voltmeter readings and a programme written in MATLAB/Simulink R2018b (MathWorks, Natick, MA, USA) [56,57]. Detailed results of this part of the research are included in [38].

## 3. Main Research Rig and Research Procedure

**Figure 5:**
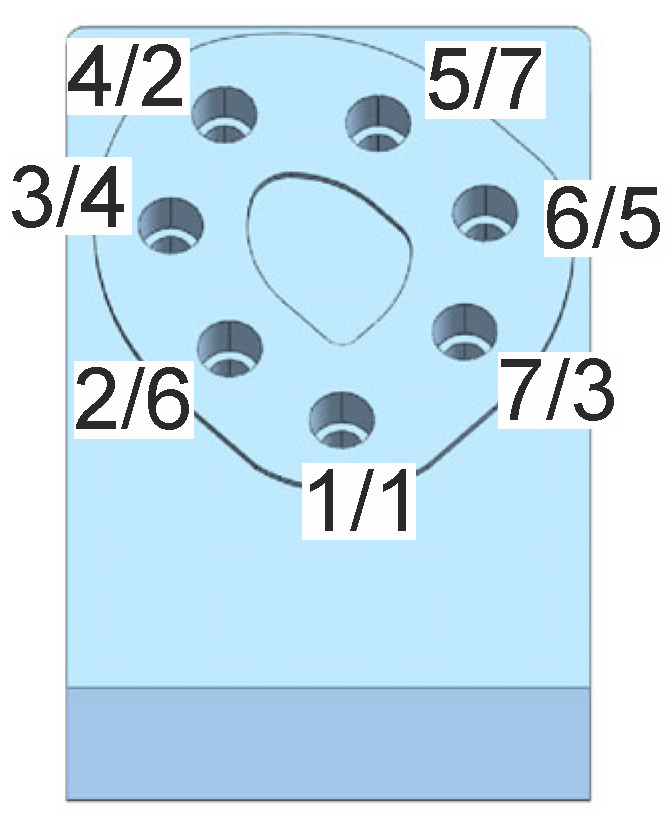
Numbering of bolt holes used in the tests and the order of preloading the bolts (added after slashes).

The bolts in the multi-bolted connection shown in Figure 1 were preloaded with a force *F* equal to 22 kN, selected using the EN 1993-1-8 standard [51]. The connection was fastened in three stages in accordance with the sequence identified in [36] as the most suitable in this case, given after the slashes next to the bolt hole numbers in Figure 5. In the first stage, the bolts were loaded to 20% of the *F* force value, in the second stage they were loaded to 60% of the *F* force value, and in the third stage they were loaded to the full *F* force value.

**Figure 6:**
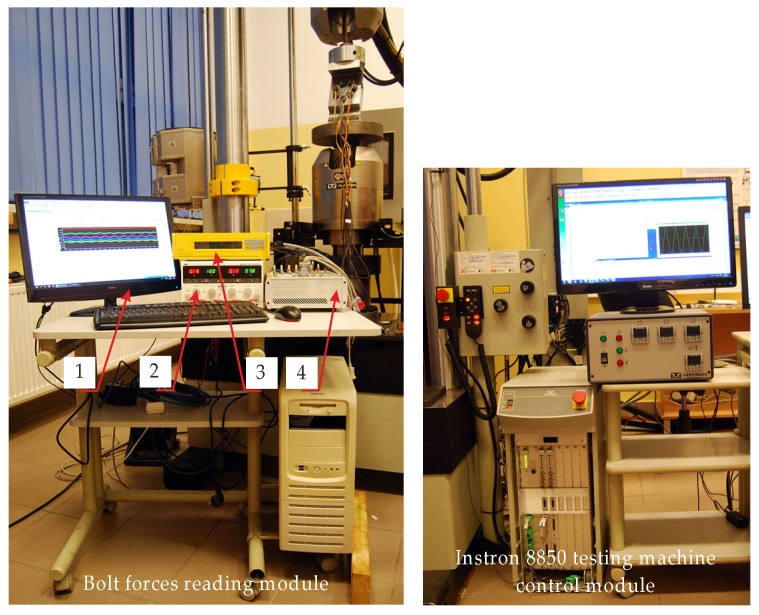
View of the main research rig (1—personal computer; 2—NDN laboratory power supply; 3—Esam Traveller CF; 4—dSPACE MicroLabBox).

The preloaded multi-bolted connection was then positioned between the upper and lower heads of the Instron 8850 servo-hydraulic testing machine (Instron GmbH, Darmstadt, Germany) using auxiliary plate supports that allow compression, thus forming the main research rig (Figure 6). The rig consists of two modules. The first one is a standard module for controlling the testing machine with which it is equipped. The tests were performed using professional testing machine software called WaveMatrix, version 1.5.318 (Instron GmbH, Darmstadt, Germany) [58]. The second one is a module for reading the force values in the bolts, composed of the following elements:

- Personal computer (PC) with the following specifications: AMD Ryzen 5 5600 G with Radeon Graphics, 3.90 GHz, 32 GB RAM, Windows 11;
- DF1743005C NDN four-channel laboratory power supply (NDN-Zbigniew Daniluk, Warsaw, Poland) [59];
- Esam Traveller CF signal conditioner amplifier system with SGA 2D plug-in card (ESA Messtechnik GmbH, Mögglingen, Germany) [60];
- dSPACE MicroLabBox—compact, integrated, all-in-one system designed for rapid control prototyping in laboratory conditions (dSPACE GmbH, Paderborn, Germany) [61].

**Figure 7:**
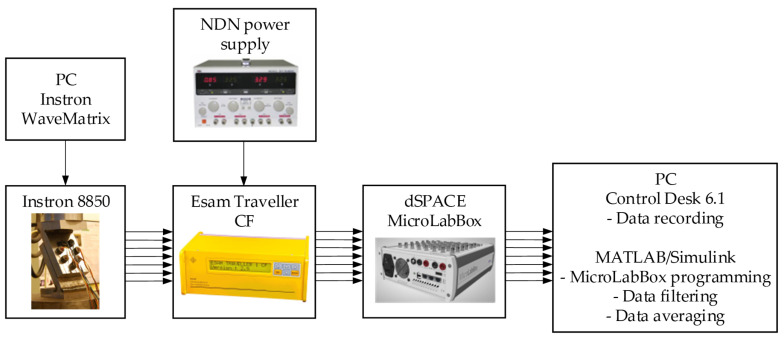
Block diagram of the test rig.

A block diagram of the entire test rig is shown in Figure 7, while a summary of the key features of the bolt forces reading module components is given in Table 1. Their detailed specifications can be found in [59,60,61].

**Figure 8:**
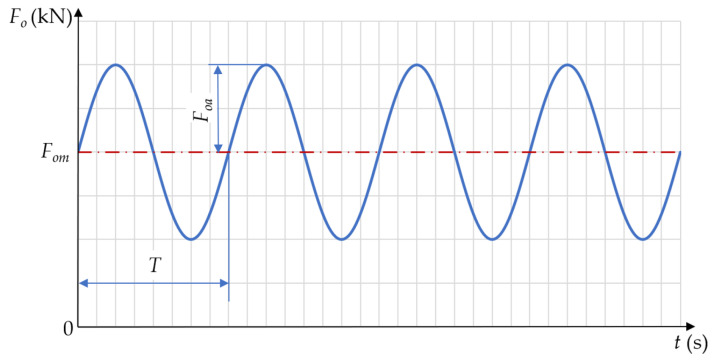
Waveform of operating force variability (*F_om_*—mean force, *F_oa_*—force amplitude, *T*—period).

Under operating conditions, the tested connection was subjected to a cyclically varying load with the general waveform shown in Figure 8. The adopted values defining specified force waveforms are collected in Table 2. The first six cycles were characterised by pulsating waves, while the last three had a reduced amplitude compared to the mean value. As a result of the applied operating loads, variable waveforms were obtained for each *i*-th bolt (for *i* = 1, 2, 3, …, 7).

## 4. Results

**Figure 9:**
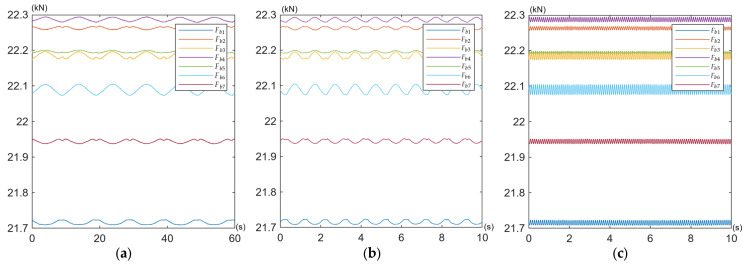
Time waveforms of working force variations in the bolts *F_bi_* during (**a**) Test No. 1; (**b**) Test No. 2; (**c**) Test No. 3.

**Figure 10:**
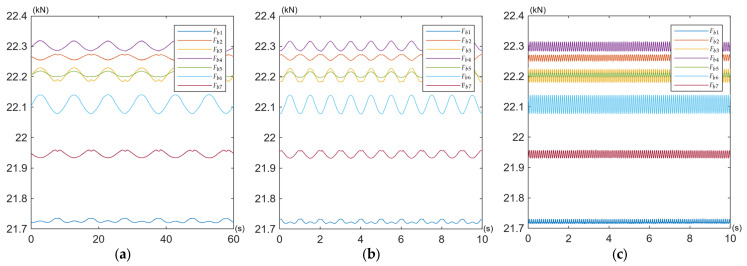
Time waveforms of working force variations in the bolts *F_bi_* during (**a**) Test No. 4; (**b**) Test No. 5; (**c**) Test No. 6.

**Figure 11:**
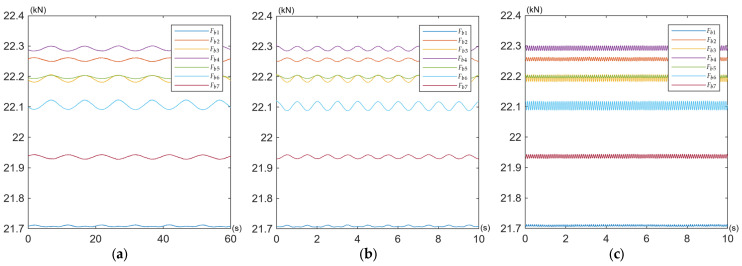
Time waveforms of working force variations in the bolts *F_bi_* during (**a**) Test No. 7; (**b**) Test No. 8; (**c**) Test No. 9.

The obtained curves of the working force variations in the bolts *F_bi_* are shown for all tests defined in Table 2 in Figure 9, Figure 10 and Figure 11.

The mean values of the forces in the bolts *F_bmi_* and their amplitudes *F_bai_* are presented in Table 3 and Table 4, respectively.

The maximum values of force in bolts *F_bmaxi_* can be determined as the sum of

(1) $$F_{bmaxi}=F_{bmi}+F_{bai}$$

where *F_bmi_* and *F_bai_* denote the mean values of the forces in the bolts and their amplitudes, respectively. The maximum force values for individual tests are given in Table 5.

## 5. Discussion

Under pulsating excitation, the time waveforms of some bolt forces exhibited deviations from the ideal sinusoidal waveform (Figure 9a,b and Figure 10a,b). This behaviour indicates variable contact stiffness between the joined components and the induction of additional bending moments in the bolts, commonly referred to as prying action [62]. This mechanism is the most probable cause of the observed phenomenon in asymmetric multi-bolted connections subjected to bending, such as the one investigated in this study. Because the connected plates are not perfectly rigid, their instantaneous centre of rotation shifts slightly during the load cycle, leading to a non-linear distribution of contact pressures. Consequently, even when the applied operating load is a perfect sine wave, its transfer to the bolts becomes non-linear due to dynamic changes in contact stiffness (e.g., localised separation or compression of the mating surfaces). This results in a distorted sinusoidal response in the bolts, often characterised by flattening at one of the peaks.

**Figure 12:**
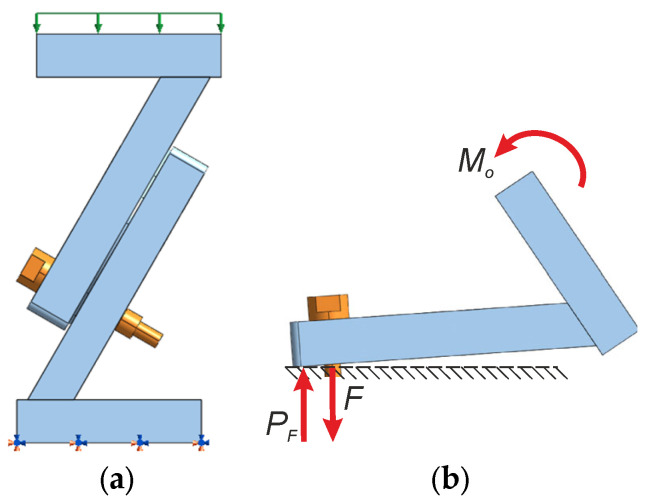
Prying action mechanism: (**a**) connection model with Bolt No. 1 loaded from above (green force vectors) and fixed from below (pink and navy blue symbols); (**b**) model position of prying force (*M_o_*—bending moment caused by operating force *F_o_*).

After the bolts had been initially tightened to 22 kN, the contact pressure was distributed over a certain area around the bolt holes. In this state, the ‘centre of rotation’ roughly coincides with the geometric centre of the figure formed by the sum of the contact areas of all the bolts. As mentioned earlier, the application of an external load on a testing machine generates a complex load condition at the contact surface: shear force, normal (compressive) force and an additional bending moment. As a result of the bending moment, the lower edge of the joined plates is pressed more firmly together, whilst the opposite edge tends to spread apart (the contact stress there decreases). Consequently, as the load increases, the pivot point shifts from the centre of the bolt arrangement towards the outer edge of the plates, which is being compressed more strongly. This edge acts as a fulcrum for the prying action. To illustrate the mechanism of the prying action, Figure 12 shows its simplified mechanical model. It depicts how the elasticity of the plates joined in the connection and the asymmetry of the bolt distribution and contact surface cause the effective centre of rotation to shift from the bolt line towards the outer edge of the plates. This shift results in the creation of a prying force *P_F_* and is responsible for the observed non-linear changes in bolt forces.

A quantitative assessment of the values of the forces collected in Table 5 can be made on the basis of the *Z* indicator defined as follows:

(2) $$Z=\frac{F-F_{bmaxi}}{F}\cdot 100$$

where *F* denotes the preload of individual bolts (equal to 22 kN, as stated at the beginning of Section 3) and *F_bmaxi_* denotes the maximum value of force in bolts.

Under the specified load conditions, the overall behaviour of the entire bolt assembly is characterised by exceptional preload stability under cyclic operating loads. All seven bolts retained their initial preload of 22 kN, with deviations not exceeding ±2% (see Table 5). In nine test variants (for different amplitudes and frequencies of external load) no relaxation or loosening was observed.

The validity of these findings is confirmed by three independent arguments:

- The measuring pathway (based on strain gauges and calibrated Wheatstone bridges) was tested during bolts calibration, which demonstrated their linear characteristics and zero hysteresis [38];
- The applied external loads (with an amplitude of 10–20 kN) remained well below the design strength of the multi-bolted connection (according to EN 1993-1-8 [51]);
- The results are repeatable for three frequencies (for periods equal to 10, 1 and 0.1 s) and two amplitudes (10 kN and 20 kN), confirming that the observed behaviour is not an artefact.

Bolts No. 1 and No. 7 behave differently from the other bolts (the forces in them are slightly smaller), but similarly to each other, despite their opposite positions in the connection. This is a direct consequence of the deliberate geometric and load asymmetry of the connection under test (resulting from the inclination of the plates relative to the base, the uneven distribution of bolts and their odd number). As shown in the prying action model (Figure 12), the effective centre of rotation shifts towards the outer edge of the plates during the load cycle. Bolts No. 1 and No. 7, located at the extreme positions relative to this shifted axis of rotation, experience a slight unloading effect (the prying force *P_F_* partially counteracts the external moment *M_o_*). The symmetry of their reactions results from the fact that both bolts lie on the same ‘prying line’ in the load plane (see Figure 1b and the numbering of the bolts in Figure 5). In contrast, bolts numbered 3–6, which are positioned closer to the centre, absorb additional compressive forces. This load redistribution model is quantitatively confirmed by the Z indicator. The lowest forces were achieved in bolts No. 1 and No. 7, which were also slightly lower than the assumed preload, by less than 2%. In the other bolts, the operating forces increased above the preload value, also by no more than 2%. These changes had no effect on bolt fatigue or on the strength and stiffness of the entire connection. The maximum forces acting on the bolts (see Table 5) remain well below the proof load of grade 8.8 bolts (equal to 35.5 kN for M10 × 1.25 bolts [48]). Cyclic variations (with an amplitude of less than ±0.1 kN) result in stress amplitudes below the fatigue limit in accordance with EN 1993-1-8 [51]. This demonstrates that a correctly preloaded, asymmetric multi-bolted connection can operate safely under cyclic operating loads without additional measures to prevent loosening—a result that has not previously been documented for asymmetrical geometries.

The quantitative data obtained, together with the minor changes in bolt forces observed, will serve as a reference point for a full publication on the validation of a systemic approach to MBCs modelling. This validation will concern preloaded systems that may be subjected to monotonic or cyclic loading, and will aim to demonstrate that the developed system model correctly represents both symmetric and highly asymmetric MBCs.

## 6. Concluding Remarks

This paper presents the results of cyclic loading tests conducted on a highly asymmetric multi-bolted connection using a custom-developed laboratory rig. While the rig’s design was introduced in a previous study [38], its application for the continuous monitoring of bolt forces under cyclic operating conditions in a geometrically and load-wise asymmetric configuration is entirely novel. This approach provides quantitative data on complex joint geometries, directly addressing the research gap identified in the Introduction.

The key findings of our studies are as follows:

1. The entire bolt assembly demonstrated remarkable stability in bolt forces, with force deviations restricted to a narrow band of ±2% relative to the initial 22 kN preload across all nine test variants;
2. Bolts No. 1 and No. 7 (located below the prying line) showed symmetric unloading, whilst the central bolts experienced slight overloading. This internal load redistribution is a predictable consequence of the connection’s geometric asymmetry and is fully consistent with the prying action mechanism;
3. The absence of preload relaxation, self-loosening phenomena, or fatigue-relevant stress amplitudes confirms that the applied cyclic operating forces have a negligible impact on the overall durability of the preloaded connection.

These results provide quantitative experimental evidence that preloaded asymmetric MBCs behave in a stable and predictable manner under cyclic loading. This dataset is of fundamental importance for validating a systemic approach to modelling MBCs and will be used in a forthcoming publication dedicated to full validation. The laboratory rig remains available for further research (e.g., at higher external load amplitudes or for fatigue testing).

Crucially, this study demonstrates that significant geometric and loading asymmetry does not compromise the structural integrity of a correctly preloaded multi-bolted connection. The minor force deviations observed should not be interpreted as a lack of dynamic response, but rather as empirical proof of the system’s robustness. This quantitative confirmation reinforces the reliability of utilising preloaded MBCs in critical structures subjected to dynamic or seismic excitations.

## Figures and Tables

**Table 1:** Key features of the bolt forces reading module components.

Measuring Component	Feature	Value
NDN laboratory power supply	Output voltage	2 × (0–30 V), 1 × (3–6 V), 1 × (8–15 V)
	Output current	2 × (0–5 A), 1 × 3 A, 1 × 1 A
	Voltage measurement accuracy	±1% + 2 digits
	Current measurement accuracy	±2% + 2 digits
Esam Traveller CF	Housing	For 16, 32 and 64 channels systems
	Number of channels	8 analogue channels per analogue board
	Data acquisition	Simultaneous
	A/D-converter	16 bit A/D-converter for each analogue channel
	Filter	Digital hardware filter for each channel
Dspace MicroLabBox	Real-time processor	Dual-core NXP (Freescale) QorIQ P5020 clocked at 2 GHz
	FPGA chip	User-programmable (Xilinx Kintex-7)
	Memory	1 GB DRAM
	Flash memory	128 MB
	Analogue inputs	24 channels, 16-bit, 1 million samples/s, range ±10 V
	Analogue outputs	16 channels, 16-bit, 1 million samples/s, range ±10 V
	Digital I/O	Over 40 channels, programmable functions

**Table 2:** Characteristics of multi-bolted connection load waveforms.

Test No.	*F_om_* (kN)	*F_oa_* (kN)	*T* (s)
1	10	10	10
2			1
3			0.1
4	20	20	10
5			1
6			0.1
7	20	10	10
8			1
9			0.1

**Table 3:** Mean values of forces in bolts *F_bmi_* for individual tests.

Test No.	*F_bm_*_1_ (kN)	*F_bm_*_2_ (kN)	*F_bm_*_3_ (kN)	*F_bm_*_4_ (kN)	*F_bm_*_5_ (kN)	*F_bm_*_6_ (kN)	*F_bm_*_7_ (kN)
1	21.716	22.263	22.185	22.287	22.196	22.090	21.944
2	21.715	22.263	22.184	22.286	22.195	22.090	21.944
3	21.715	22.263	22.184	22.287	22.195	22.090	21.944
4	21.726	22.264	22.206	22.300	22.208	22.110	21.946
5	21.724	22.264	22.204	22.300	22.206	22.108	21.945
6	21.721	22.262	22.201	22.297	22.204	22.107	21.943
7	21.708	22.255	22.193	22.292	22.198	22.106	21.936
8	21.708	22.256	22.194	22.292	22.199	22.103	21.937
9	21.709	22.256	22.194	22.292	22.198	22.103	21.937

**Table 4:** Force amplitude values *F_bai_* for individual tests.

Test No.	*F_ba_*_1_ (kN)	*F_ba_*_2_ (kN)	*F_ba_*_3_ (kN)	*F_ba_*_4_ (kN)	*F_ba_*_5_ (kN)	*F_ba_*_6_ (kN)	*F_ba_*_7_ (kN)
1	0.011	0.007	0.012	0.009	0.005	0.017	0.008
2	0.008	0.006	0.011	0.008	0.005	0.016	0.007
3	0.008	0.006	0.011	0.009	0.004	0.016	0.008
4	0.009	0.011	0.024	0.018	0.012	0.032	0.014
5	0.008	0.010	0.023	0.017	0.010	0.032	0.014
6	0.009	0.016	0.025	0.017	0.009	0.032	0.014
7	0.004	0.008	0.013	0.010	0.007	0.017	0.009
8	0.004	0.007	0.014	0.012	0.006	0.018	0.008
9	0.004	0.008	0.013	0.009	0.005	0.016	0.008

**Table 5:** Maximum force values *F_bmaxi_* for individual tests.

Test No.	*F_bmax_*_1_ (kN)	*F_bmax_*_2_ (kN)	*F_bmax_*_3_ (kN)	*F_bmax_*_4_ (kN)	*F_bmax_*_5_ (kN)	*F_bmax_*_6_ (kN)	*F_bmax_*_7_ (kN)
1	21.727	22.270	22.197	22.296	22.201	22.107	21.952
2	21.723	22.269	22.195	22.294	22.200	22.106	21.951
3	21.723	22.269	22.195	22.296	22.199	22.106	21.952
4	21.735	22.275	22.230	22.318	22.220	22.142	21.960
5	21.732	22.274	22.227	22.317	22.216	22.140	21.959
6	21.730	22.278	22.226	22.314	22.213	22.139	21.957
7	21.712	22.263	22.206	22.302	22.205	22.123	21.945
8	21.712	22.263	22.208	22.304	22.205	22.121	21.945
9	21.713	22.264	22.207	22.301	22.203	22.119	21.945

## Data Availability

The original contributions presented in this study are included in the article. Further inquiries can be directed to the corresponding author.

## Abbreviations

The following abbreviations are used in this paper:

MBCs	Multi-bolted connections
PC	Personal computer
SBCs	Single-bolted connections
